# Supplementary material for: Improving the working environment for the delivery of safe surgical care in the UK: a qualitative cross-sectional analysis
Source: BMJ Open. 2019 Jan 24;9(1):e023476. doi: 10.1136/bmjopen-2018-023476 (PMC6347853; doi:10.1136/bmjopen-2018-023476)
Supplement: Author's manuscript [file bmjopen-2018-023476.draft_revisions.pdf]

BMJ Open is committed to open peer review. As part of this commitment we make the peer review history of every article we publish publicly available.

When an article is published we post the peer reviewers' comments and the authors' responses online. We also post the versions of the paper that were used during peer review. These are the versions that the peer review comments apply to.

The versions of the paper that follow are the versions that were submitted during the peer review process. They are not the versions of record or the final published versions. They should not be cited or distributed as the published version of this manuscript.

BMJ Open is an open access journal and the full, final, typeset and author-corrected version of record of the manuscript is available on our site with no access controls, subscription charges or pay-per-view fees (http://bmjopen.bmj.com).

If you have any questions on BMJ Open's open peer review process please email info.bmjopen@bmj.com

BMJ Open

# Improving the working environment for the delivery of safe surgical care in the United Kingdom: A cross-sectional analysis

| | |
|---|---|
| Journal: | *BMJ Open* |
| Manuscript ID | bmjopen-2018-023476 |
| Article Type: | Research |
| Date Submitted by the Author: | 08-Apr-2018 |
| Complete List of Authors: | Baggaley, Alice; Homerton University Hospital NHS Foundation Trust,<br>Robb, Lydia; Royal Infirmary of Edinburgh<br>McGregor, Richard ; Royal College of Surgeons in Edinburgh Training Commitee<br>Paterson-Brown, Simon; Royal Infirmary of Edinburgh |
| Keywords: | MEDICAL EDUCATION & TRAINING, Organisation of health services < HEALTH SERVICES ADMINISTRATION & MANAGEMENT, SURGERY |
| | |

SCHOLARONE™
Manuscripts

For peer review only

# Improving the working environment for the delivery of safe surgical care in the United Kingdom: A cross-sectional analysis

For peer review only

Baggaley[1], L. Robb[2], S Paterson-Brown[3], R. J McGregor[3]

1. Homerton University Hospital, Hackney, London, UK, E96SR
2. Victoria Hospital, Kirkcaldy, Fife, UK, KY25AH
3. Edinburgh Royal Infirmary, Edinburgh, UK, EH1645A

Author correspondence: Richard.mcgregor@ed.ac.uk

Word Count: 2980

**ABSTRACT**

**Objectives:** The aim of this study was to identify current problems and potential solutions to improve the working environment for the delivery of safe surgical care in the United Kingdom.

**Design:** Prospective, questionnaire-based cross-sectional study.

**Setting/Participants:** Following validation, a 5 item electronic questionnaire was distributed to postgraduate local education and training board (LETB) distribution lists, the Royal College of Surgeons of Edinburgh (RCSEd) mailing lists and other trainee organisations. Throughout the 13 week study period the survey was also published on a number of social media platforms.

**Results**: A total of 505 responders completed the survey, of which 35% were consultants, 30% Foundation Doctors, 17% speciality trainees, 11% specialty doctors, 5% core trainees, and 1% surgical nurse practitioners. A total of 2238 free text answers detailed specific actions to improve the working environment. These responses were individually coded and grouped into 9 categories. Common themes emerged across all grades including: increased front line staff; a return to a “firm” structure to improve team continuity; greater senior support; and improved hospital facilities to help staff rest and recuperate.

**Conclusions**: The results of this study have identified a number of key areas that if addressed, can improve the environment for the delivery of safer surgical care. Many responses were interconnected and reflect the unprecedented strain that the National Health Service (NHS) is currently facing. While unlimited funding remains unrealistic, many of the suggestions could be implemented in a relatively cost neutral fashion and include insightful ideas for remodeling or restructuring the workforce to improve the efficiency of the surgical team. The

For peer review only

findings of this study formed the basis of a set of recommendations published by the RCSEd as a discussion paper.

**Key Words: Training, questionnaire, patient safety**

**Article Summary**

**Strengths and limitations of this study**

1. This study describes the detailed perspectives and opinions of frontline NHS staff regarding current problems and potential solutions for improving the delivery of safe surgical care in the UK at a time of unprecedented strain.
2. The wide distribution of this survey throughout the United Kingdom and response rates across the training/non-training grades helps to mitigate against sub-group bias and generates a depth and richness to the answers.
3. Whilst some groups had higher response rates than others this is likely explained via varying degrees of penetration and distribution across the electronic mailing lists.
4. Despite the brevity of the survey we found a higher than expected incompletion rate (46%), however the demographics of those who did not fully complete the survey were comparable to those that did complete the survey, eliminating a potential completion bias of the respondents.
5. The extended surgical team is becoming ever more important when considering the multi-disciplinary approach to the delivery of acute surgical services and therefore the limited responses from this group of staff is a weakness.

For peer review only

**INTRODUCTION**

The delivery of safe surgical care within the United Kingdom is a complex and multi-faceted issue. To date, there has been limited direct engagement with the stakeholders on the front line of acute surgical services to ascertain what they feel are the current problems in delivering safe surgical care and the potential solutions. A number of themes emerged from this study, including working patterns, team support and structure, and staff facilities.

As a piece of health and safety legislation, the European Working Time Regulations (EWTR) is intended to reduce fatigue in doctors and improve both their own safety and that of their patients. A number of organisations have expressed support for the EWTR, although often noting that there have been problems with its implementation. Health Education England (HEE) expressed the view that any increase in working hours could be dangerous to patients and doctors, while the Royal College of Obstetricians and Gynaecologists viewed the EWTR as beneficial in the context of supporting safe working practices. They all however recognise that it has had consequences, the most important of which appears to be the reduction in the available hours for training for junior doctors. It is also possible that as a measure to help combat fatigue, the necessary introduction of shift patterns may also have had an unintended consequence on patient care related to a reduction in continuity of care. Training programs find it hard to provide the continuous rest required for the EWTR without utilising full shift rotas. This means that fewer trainees are available but are much busier and have to look after larger numbers of patients. The latter is particularly true in

For peer review only

acute medicine where the medical registrar is under constant pressure, another contribution to recruitment difficulties.

Finding the balance between service provision and training has been an interminable challenge for the NHS. Innovative solutions have been suggested and the authors point to the highly successful report commissioned by HEE – *Better Training, Better Care*[1]. This programme aimed to improve the quality of training by enabling the key recommendations from Sir John Temple's *Time for Training*[2] and Professor John Collins' *Foundation for Excellence* reports[3]. A pilot project[4] was run in Leeds and York that modified the rotas to maximise the potential time for training, and *"100% of the trainees at Leeds and York have reported more confidence in their work since the pilot and 83% agree, or strongly agree, that they have benefitted from the change in rota. The pilot saw an increase in productivity with weekday activity increasing by 37.7%, weekend activity rising by 29.1% and night shift activity by 22.1%."*

While the health and well-being of the work-force underpins a small piece of the overall specialty of Human Factors and Ergonomics in health care, which in turn ties into patient safety, it is intrinsically linked to team work and team performance. What's more, there is a vast array of literature out-with healthcare, and increasingly within, which focuses upon the critical relationship between non-technical skills, team performance, and improved clinical outcome[5]. The intimate link between human error and patient safety had been placed firmly under the spotlight by a number of key studies that estimate that approximately 10% of all hospital admissions result in an adverse event[6]. This was directly responsible for the introduction of variously titled 'Patient Safety' organisations around the world and the development of 'bundles of care' for standardizing

For peer review only

protocols for a number of procedures and the World Health Organisation's 19-item Surgical Safety Checklist[7]. This cross-sectional study aimed to identify the fundamental areas to be addressed in order to improve the working environment for the delivery of safe surgical care.

## METHODS

A prospective cross-sectional qualitative online survey was created using 'Survey Monkey'. Participants were first asked to provide basic demographics including; gender (male, female); grade (consultant, registrar, speciality doctor, core trainee, foundation year trainee, nurse, other); location (England, Wales, Scotland, Northern Ireland); and hospital setting (large city hospital, district general hospital, remote and rural hospital). Thereafter, sequential white-space boxes allowed for 5 free-text responses (up to 4000 characters each) to the following open question:

*"In the delivery of safe surgical care, what are the 5 most important things which would improve the workplace environment* <u>*for you*</u>*?"*

The survey was developed by the Royal College of Surgeons of Edinburgh Short Life Working Group (SLWG) and approved by the Patient Safety Board, with the explicit aim to elicit as broad a response as possible, preferably from the multi-disciplinary team as well as patients. The survey was distributed to members of the surgical teams in hospitals across the UK, using the RSCEd contact lists and appointing representatives within the SLWG. These representatives for foundation year, core and speciality trainees could themselves utilise deanery e-

For peer review only

mail lists and social media groups. The survey was open for responses for 13 weeks between 8th February to 14th May 2016.

Two members of the SLWG independently read each of the text responses and created codes as new topics were broached (appendix 1). The final 92 codes were then discussed with the SLWG and grouped into nine broad categories (fig. 1). Analysis of the results looked at response by grade of doctor, as well as the distribution of responses within each broad category.

**Patient Involvement**

The questionnaire used in this study was approved by the RCSEd Patient Safety Board.

**RESULTS**

A total of 932 people started the survey, however only 505 responders proceeded to complete at least one free text response, resulting in a 54% completion rate. Those 427 remaining responders who did not meet the inclusion criteria of entering at least one free text answer were excluded from further analysis. The 505 participants generated a total of 2238 individual free text responses. The demographics of the respondents are shown in figure 2.

The greatest number of free text responses was generated from consultants and foundation year trainees (801 and 651 respectively). Despite distributing the survey amongst patient safety groups, no patients took part. Only 6 nurse practitioners completed any free text responses and their results

For peer review only

are not presented here due to insufficient numbers. Figure 3 shows the overall responses across each of the nine broad categories, regardless of grade. The vast majority of responses relate to resources, and were further subdivided as either staff or non-staff related resources. The next most frequently recurring responses fell into the communication and teamwork, working conditions, and support categories.

There were a high number of recurring responses throughout the survey, and Table 1 lists the top five from each grade. The need for more staff was an area of concern identified across all grades. This included not only more doctors, but also increased numbers of all members of the surgical team. Interestingly the need for more administrative and secretarial staff was mentioned regularly, which likely represents the high burden of 'paperwork' in the modern working environment.

| | |
|---|---|
| **Consultant** | Staff<br>Return to firm approach<br>Improved access to imaging/diagnostic services/ interventional radiology<br>Improved access to emergency theatre<br>Ring-fenced patients |
| **Specialty Registrar** | Staff<br>Improved facilities<br>Return to firm approach<br>Improved IT systems<br>Improved handover |
| **Core Trainee** | Staff<br>Senior support<br>Support from other specialities<br>More time in theatre |

| | |
|---|---|
| | Improved facilities |
| **Specialty Doctor** | Staff<br>Senior support<br>Teaching/training<br>Adequate equipment<br>Improved facilities |
| **Foundation Doctor** | Staff<br>Senior support<br>Teaching/training<br>Improved IT systems<br>Better communication |

**Table 1 – Top 5 responses by grade**

*Consultants*

Consultants provided 801 individual free-text responses; over half of these related to resources, both staff and non-staff related (fig. 4). The comments regarding non-staff resources included suggestions such as "better information technology systems", "more beds" and "better availability of imaging/diagnostic services". The most frequent individual code pertained to increased staff, which included medical and surgical axillary staff but particularly noted the need for more nursing staff.

Within the working conditions category over half of the responses pertained to "continuity of team members" and "returning to a 'firm' approach". For the support category, consultants mostly referred to support from hospital management, as opposed to that of senior colleagues as suggested by the more junior grades. Within the communication and teamwork category, consultants suggested better communication skills, improved teamwork and improved


For peer review only - http://bmjopen.bmj.com/site/about/guidelines.xhtml

morale was required to enhance the working environment. For systems improvements, the need for less bureaucracy, less focus on service provision and a reduction in use of protocols was suggested. Only the foundation year doctors mentioned the latter suggestion in any great number. For the continuity of care category, the popular responses were continuity of patient care, and the need to operate on ones' own patients. This links to the frequently suggested need to return to a 'firm' structure in order to improve continuity of team members.

*Registrars and Core Trainees*

Collectively this group provided 434 free text responses, with the largest category being non-staff resources (22% compared with 37% of consultant responses) (fig. 5). Within the working conditions category, continuity of team members and improvement to the rota were both popular codes. However the most frequent code was the suggestion of improvement to facilities such as a hospital mess, increased parking, office space, changing rooms, showers and out of hours catering. These suggestions were common across all grades. Registrars felt improvements to handovers were important, more so than any other grade. Perhaps a reflection of the training posts that the registrars and core trainees hold, another common theme was that of improvement to appraisals and feedback, as well as an increase in both quality and quantity of teaching.

*Specialty and Associate Specialty Doctors*

For peer review only

Similarly to consultants, around one third of specialty doctors' responses related to non-staff resources such as adequate equipment and improved computer systems. Of all the grades, specialty doctors provided the highest number of responses within the training and education category, which mostly related to career progression and access to, and recognition of, training. Specialty doctors also sought greater support from their seniors as well as hospital management. Subjectively, there appeared to be a sense of frustration at being undervalued within the system. This is of particular importance as progression into training posts declines, making recruitment and retention of specialty doctors even more vital.

*Foundation Year Doctors*

Foundation doctors comprised the second largest group after consultants, with 651 free text responses generated (fig. 7). Compared with the other grade groups, foundation year doctors provided the lowest number of responses regarding non-staff resources, but the highest percentage of responses regarding support (18%), which frequently involved suggesting more support from their senior colleagues. Compared with consultants, foundation year doctors were much more concerned with communication and teamwork (20% vs. 9%), and suggested that improved handovers, better team cohesion, and clearer plans from their consultants regarding patients might improve the working environment for safe surgical care.

Within the category staff resources, foundation year doctors provided the highest total number of responses and tended to seek more staff to support their



For peer review only

ward roles, such as phlebotomists, healthcare assistants and physician associates, as well as more 'juniors' to facilitate the staffing of a full rota. Certainly the recognition of the increasing load of non-clinical ward tasks has led to expansion of the 'wider surgical team', with the recruitment of advance nurse practitioners, prescribing pharmacists and physician associates.

| Box 1 **Representative qualitative comments from respondents** |
|---|
| • "I want to be able to take annual leave and not be bullied by management to work to cover shifts."<br>• " With better staffing we could reallocate tasks, reducing the hours spent on duties such as phlebotomy, cannulas and discharge letters."<br>• "We ought to have a senior-led ward round at least once per day."<br>• "Better out-of-hours access to urgent radiology would improve surgical safety."<br>• There should be on-call facilities so night staff can rest if they are not busy and can sleep in the morning if they are too tired to drive home after night shift."<br>• "Involvement of clinicians in workplace decisions and changes - e.g. changes to gloves/needles/gowns to save money, ignoring the concerns of those using them."<br>• "More value put on support staff such as secretaries and ward clerks to ensure administrative processes work reliably." |

For peer review only

## DISCUSSION

Many responses were interconnected and reflect the unprecedented pressure that the National Health Service (NHS) is currently under. For example, it is easy to see how 'rota gaps' left by too few staff can lead to low morale, poor communication, lack of continuity of care for patients and reduced teamwork. While unlimited funding remains unrealistic, many of the suggestions from front line staff could be implemented in a relatively cost-neutral fashion. For instance, the use of smart rotas that aim to increase daylight training time and return to a firm structure[8] could improve patient safety, morale and training, and in turn improve recruitment and retention within surgery.

### *The Need for Structured Senior Support*

There was a very strong message from the free text answers that better supervision and support for both foundation doctors and speciality trainees would improve overall safety. It is postulated, quite reasonably, that better supervision and support would lead to less stressed juniors who will not only make fewer mistakes, but will have more resilience, higher productivity and less absenteeism[9]. This in turn will permit better patient care and more meaningful learning to take place in line with the new GMC Standards for Excellence[10]. Communication and a sense of belonging to 'the team' would alleviate such perceptions of lack of support. Both consultants and specialty trainees might consider a 'mid-day catch-up' with their juniors to provide advice and moral support, phoning the junior doctor in between cases could provide an alternative if seniors were unable to leave theatre to meet at mid-day.

For peer review only - http://bmjopen.bmj.com/site/about/guidelines.xhtml

Improving overall support for all trainees is clearly a major factor underpinning both a better working environment but also improved patient safety. This requires a significant change in service delivery so that consultants and specialty trainees are free from all elective activity when 'on-call' and can therefore become more involved during the day in the running of the surgical wards and supporting the more junior doctors and other members of the surgical team. Many units now do offer this service and have found that this has allowed a partial return to the old 'firm' structure where the same team looks after a group of patients for several days in a row.  This requires a significant change in how units and hospitals function, re-organisation of clinics, operating sessions and ward rounds, as well as identification of those surgeons who are not in theatre and might therefore be available to help out with problems on the wards when they arise during the day. The introduction of experienced 'surgical nurse practitioners' has greatly facilitated such activities, providing someone who knows the unit system, where senior staff can be found on a daily basis, as well as the unit protocols for management of specific conditions. While such persons require additional funding, those units who have invested in them have reaped rewards in improved efficiency, patient care and trainee support. In some specialties such as Urology, experienced nurses can provide front line urgent clinics, thereby reducing admissions and workload for the trainee doctors[11]. Similarly, consultant led emergency 'hot clinics' have been shown to result in a significant reduction in emergency admissions and hospital stay, which in turn has an effect on reducing overall workload perception[12].

***The Challenge of Working hours: safety, service, and time for training***

For peer review only - http://bmjopen.bmj.com/site/about/guidelines.xhtml

Interestingly, there were very few comments on the number of hours worked. However, linked to the numerous comments surrounding the need for more staff lies the challenge of creating rotas that comply with the European Working Time Regulations while at the same time facilitate excellence in training and safe surgical care. A key document published in March of 2014 highlighted the Impact of the EWTR on the NHS and Health Professionals[13].

Overall, while it appears clear that patient safety is improved by ensuring that doctors do not work long hours, the current issues relate to arduous night shifts, often with inadequate senior support, that bring their own health-related problems. Staff working such intense hours overnight (both medical and nursing), have to drive home the following day, and there have been many reports of fatalities due to tiredness.

***A Focus on Human Factors***

Following the controversial introduction of the new Junior Doctors contract in 2016, progression into higher training is at an all-time low. A survey in 2017 by the UK Foundation Programme Office found only 42.6% of Foundation Year 2 doctors planned to go directly into training[14]. In the light of the result of our survey, building a resilient workforce must be an absolute priority for the future well-being of the NHS. This will be greatly helped by diversifying our care-provider model to involve an alternative workforce with appropriate skill mix to supplement junior doctors, particularly during peaks of service demands and in areas with chronic shortages in junior medical staffing, allowing junior doctors the time to have more meaningful patient encounters and reduce unnecessary and stressful interruptions in tasks.

For peer review only - http://bmjopen.bmj.com/site/about/guidelines.xhtml

***Hospital Facilities***

This study has shown that there remains inadequate access to hot food and appropriate facilities where staff can relax during their breaks (if they are fortunate enough to get them) without meeting patients or their relatives. Lack of a common area, such as a hospital mess, makes it more difficult for consultants and trainees to have "catch up meetings", where advice and moral support can be provided. Improvement in the hospital working environment has been shown to improve safety and quality in hospital care, whilst simultaneous increasing patient satisfaction[15].

**Table 2 – Summary of RCSEd key recommendations**

| |
|---|
| RECOMMENDATION 1: **Establish structured senior support** |
| RECOMMENDATION 2: **Reintroduce the hospital mess** |
| RECOMMENDATION 3: **Intelligent design of rotas** |
| RECOMMENDATION 4: **Streamline and reorganize the overall workload to prioritise core clinical duties and create an integrated multidisciplinary surgical team** |
| RECOMMENDATION 5: **Recognise that better training delivers better care** |
| RECOMMENDATION 6: **Promote human factors training** |
| RECOMMENDATION 7: **Support and Training the Trainers** |

**CONCLUSIONS**

The results of the survey were used as a springboard for the RCSEd recommendations (table 2), published as a discussion paper, that hope to

For peer review only

improve patient safety, staff morale and retention[16]. To those working in a significant proportion of surgical units, many of these recommendations will not seem surprising. Importantly, this survey provides qualitative evidence about what front line staff feel are the most pressing issues that need to be addressed in order to improve the surgical working environment. In this current climate it is more important than ever to find ways to improve patient safety within surgery and create a healthy surgical workforce for the future.

**Footnotes**

**Author Statement:** The SLWG conceived and designed the study. Survey designed by SLWG and approved by PSB of RCSEd. Survey distributed by AB, LR and RJM. AB and LR collected the data. AB and LR analysed the data, allocated prospective coding to each response. Data analysis and coding approved by SLWG. All authors were responsible for compiling the manuscript and approving the final article. AB and LR contributed equally and therefore are joint first authors.

**Funding:** This research received no specific grant from any funding agency in the public, commercial or not-for-profit sectors.

**Conflicts of Interests**: All authors have completed the ICMJE uniform disclosure form at www.icmje.org/coi_disclosure.pdf and declare: this study was commissioned by the Office Bearers of RCSEd and conducted on behalf of the RCSEd; no financial relationships with any organisations that might have an interest in the submitted work in the previous three years. SPB and RJM were RCSEd Council Members at the time of the study. SPB is chair of RCSEd Patient

For peer review only

Safety Board at the time of writing.

**Data Sharing Statement:** Respondent-level data are available from the corresponding author at richard.mcgregor@ed.ac.uk. The presented data are anonymised with low risk of identification.

For peer review only

## References

1. Better Training, Better Care. Health Education England. http://hee.nhs.uk/wp-content/blogs.dir/321/files/2012/09/Matrix-Knowledge-Better-Training-Better-Care.pdf ).
2. Temple J. *Time for Training.* London: MEE; 2010
3. Collins J. Foundation for excellence. An evaluation of the foundation programme. MEE; 2010.
4. Royal College of Surgeons of England. Improving Surgical Training. Proposal for a pilot surgical training programme. Oct 2015 www.rcsengl.ac.uk
5. Yule S and Paterson-Brown S. Surgeons' non-technical skills. In: ed Sanchez JA. Patient Safety. Surgical Clinics of North America. Elsevier Inc. 2012 pp37-50
6. Anderson O, Davis R, Hanna GB, and Vincent C. Surgical adverse events: a systematic review. AJS. Elsevier Inc; 2013 Aug 1;206(2):253–62
7. Haynes AB, Weiser TG, Berry WR, Lipsitz SR, Breizat AH, Dellinger EP, Herbosa T, Joseph S, Kibatala PL, Lapitan MC, Merry AF, Moorthy K, Reznick RK, Taylor B, Gawande AA. A surgical safety checklist to reduce


For peer review only - http://bmjopen.bmj.com/site/about/guidelines.xhtml

morbidity and mortality in a global population. NEJM 2009;360(5): 491-499

8. Professionalism Compliance Analysis Tool (PCAT) for Rotas, Qualitative Semi-structured Interviews of 23 Trainees in Scotland were conducted in 2014 to establish recurring themes of "Good Rotas". Scottish Clinical Leadership Fellows. See appendix 4 for more details. 8
9. A.J. Oswald et al. Happiness and Productivity. Journal of Labour Economics 2014; 33:(4): 789-822
10. General Medical Council (GMC). Promoting Excellence: Standards for Medical Education and Training. 2016. www.gmc-uk.org
11. Kaur K, Nevin D, Musgrove G. The Urology Rapid Access Unit (URAU): Tackling Winter Pressures - A 12-month Nurse Led Pilot. British Association of Urological Nurses. 2016
12. Th'ng F, Skouras C, Paterson-Brown A, Ravindran R, Lamb P, de Beaux A, Paterson-Brown S, Mole DJ. Emergency general surgery 'Hot Clinics' reduce admission rates and duration of inpatient stay. Frontline Gastroenterology 2015; 0:1-9. Doi: 10.1136/flgastro-2015-100634
13. The Implementation of the Working Time Directive, and its Impact on the NHS and Health Professionals: Report of the Independent Working Time Regulations Taskforce to the Department of Health (March 2014).
14. The Foundation Programme Career Destination Report, 2017. http://www.foundationprogramme.nhs.uk/news/story/f2-career-destination-survey-2017
15. Aiken H, Sermeus W, Van den Heede K et al. Patient safety, satisfaction, and quality of hospital care: cross sectional surveys of nurses and patients

For peer review only

in 12 countries in Europe and the Unite States. BMJ 2012:344:e1717

16. Paterson-Brown S, McGregor R, Robb L, Baggaley A et al. Improving the working environment for safe surgical care. A discussion paper from the Royal College of Surgeons of Edinburgh. 2017 https://www.rcsed.ac.uk/media/415574/rcsed-working-environment-web3.pdf

For peer review only

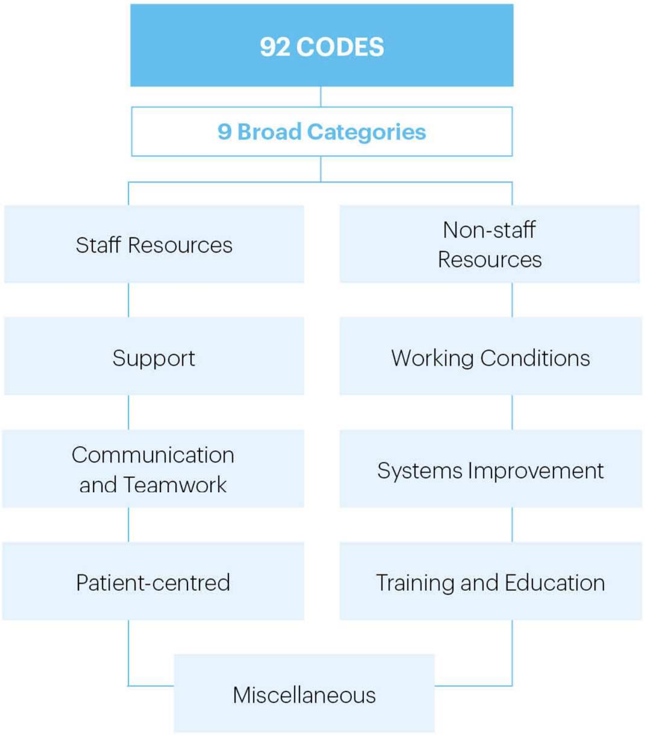


Figure 1 – Coding categories

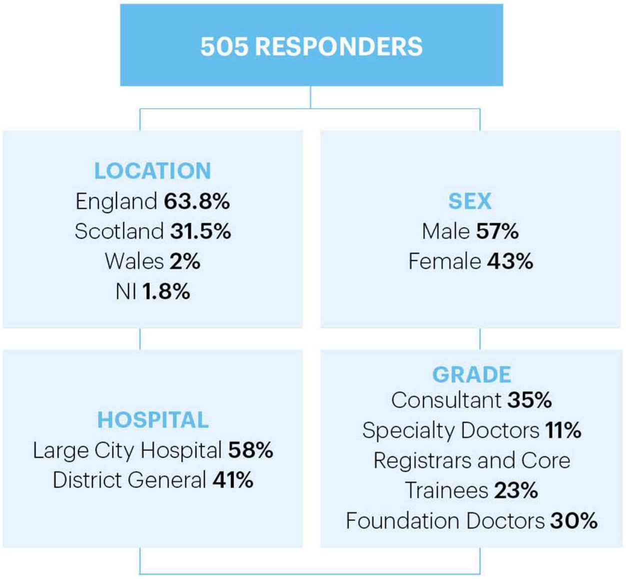


Figure 2 – Breakdown of responses

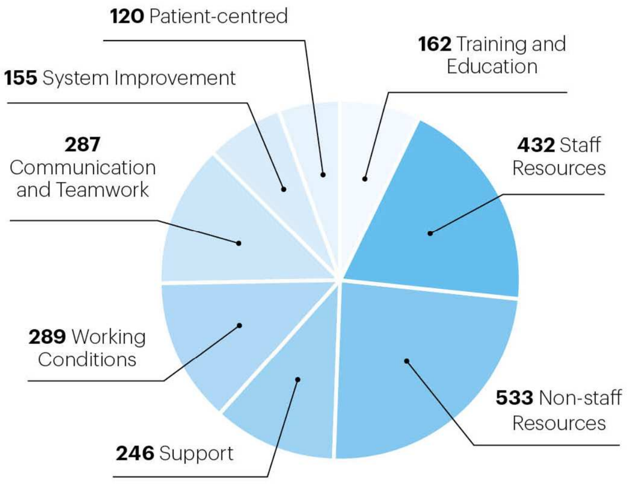


Figure 3 – Responses per category

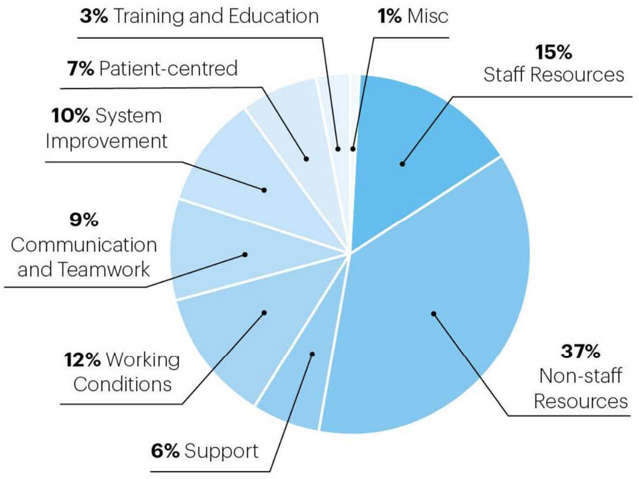


Figure 4 – Consultant responses

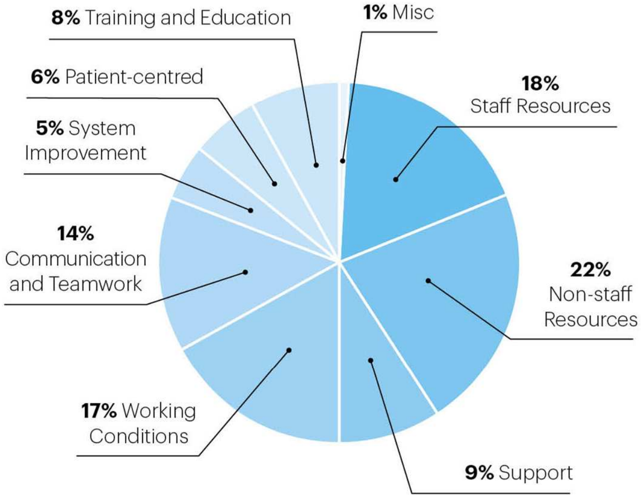


Figure 5- Registrar and Core Trainee responses

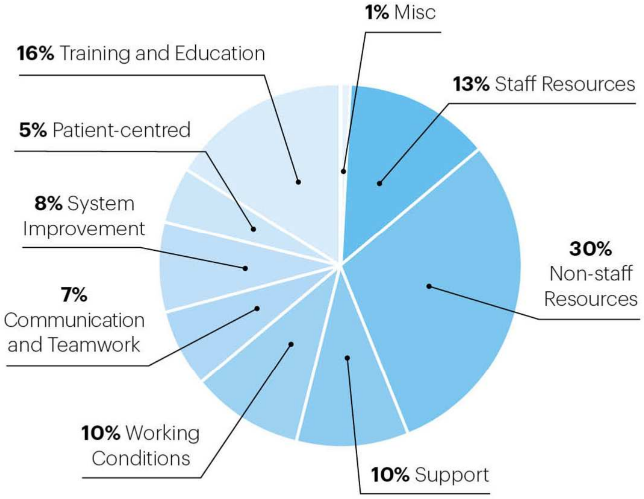


Figure 6 – Specialty doctor responses

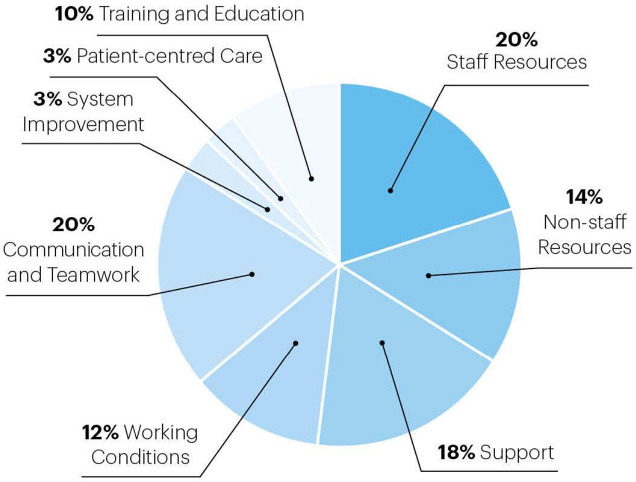


Figure 7 – Foundation year responses

# Reporting checklist for qualitative study.

Based on the SRQR guidelines.

## Instructions to authors

Complete this checklist by entering the page numbers from your manuscript where readers will find each of the items listed below.

Your article may not currently address all the items on the checklist. Please modify your text to include the missing information. If you are certain that an item does not apply, please write "n/a" and provide a short explanation.

Upload your completed checklist as an extra file when you submit to a journal.

In your methods section, say that you used the SRQR reporting guidelines, and cite them as:

O'Brien BC, Harris IB, Beckman TJ, Reed DA, Cook DA. Standards for reporting qualitative research: a synthesis of recommendations. Acad Med. 2014;89(9):1245-1251.

| | | Reporting Item | Page Number |
|---|---|---|---|
| | #1 | Concise description of the nature and topic of the study identifying the study as qualitative or indicating the approach (e.g. ethnography, grounded theory) or data collection methods (e.g. interview, focus group) is recommended | 1 |
| | #2 | Summary of the key elements of the study using the abstract format of the intended publication; typically includes background, purpose, methods, results and conclusions | 1 |
| Problem formulation | #3 | Description and signifcance of the problem / phenomenon studied: review of relevant theory and empirical work; problem statement | 4 |
| Purpose or research question | #4 | Purpose of the study and specific objectives or questions | 5 |
| Qualitative approach and research paradigm | #5 | Qualitative approach (e.g. ethnography, grounded theory, case study, phenomenolgy, narrative research) and | 6 |

For peer review only

| | | | |
|---|---|---|---|
| | | guiding theory if appropriate; identifying the research paradigm (e.g. postpositivist, constructivist / interpretivist) is also recommended; rationale. The rationale should briefly discuss the justification for choosing that theory, approach, method or technique rather than other options available; the assumptions and limitations implicit in those choices and how those choices influence study conclusions and transferability. As appropriate the rationale for several items might be discussed together. | |
| Researcher characteristics and reflexivity | #6 | Researchers' characteristics that may influence the research, including personal attributes, qualifications / experience, relationship with participants, assumptions and / or presuppositions; potential or actual interaction between researchers' characteristics and the research questions, approach, methods, results and / or transferability | n/a |
| Context | #7 | Setting / site and salient contextual factors; rationale | 6 |
| Sampling strategy | #8 | How and why research participants, documents, or events were selected; criteria for deciding when no further sampling was necessary (e.g. sampling saturation); rationale | 6 |
| Ethical issues pertaining to human subjects | #9 | Documentation of approval by an appropriate ethics review board and participant consent, or explanation for lack thereof; other confidentiality and data security issues | 6 |
| Data collection methods | #10 | Types of data collected; details of data collection procedures including (as appropriate) start and stop dates of data collection and analysis, iterative process, triangulation of sources / methods, and modification of procedures in response to evolving study findings; rationale | 6 |
| Data collection instruments and technologies | #11 | Description of instruments (e.g. interview guides, questionnaires) and devices (e.g. audio recorders) used for data collection; if / how the instruments(s) changed over the course of the study | 6 |
| Units of study | #12 | Number and relevant characteristics of participants, documents, or events included in the study; level of | 6 |

For peer review only

| | | | |
|---|---|---|---|
| | | participation (could be reported in results) | |
| Data processing | #13 | Methods for processing data prior to and during analysis, including transcription, data entry, data management and security, verification of data integrity, data coding, and anonymisation / deidentification of excerpts | 7 |
| Data analysis | #14 | Process by which inferences, themes, etc. were identified and developed, including the researchers involved in data analysis; usually references a specific paradigm or approach; rationale | 7 |
| Techniques to enhance trustworthiness | #15 | Techniques to enhance trustworthiness and credibility of data analysis (e.g. member checking, audit trail, triangulation); rationale | 7 |
| Syntheses and interpretation | #16 | Main findings (e.g. interpretations, inferences, and themes); might include development of a theory or model, or integration with prior research or theory | 7 |
| Links to empirical data | #17 | Evidence (e.g. quotes, field notes, text excerpts, photographs) to substantiate analytic findings | 12 |
| Intergration with prior work, implications, transferability and contribution(s) to the field | #18 | Short summary of main findings; explanation of how findings and conclusions connect to, support, elaborate on, or challenge conclusions of earlier scholarship; discussion of scope of application / generalizability; identification of unique contributions(s) to scholarship in a discipline or field | 13 |
| Limitations | #19 | Trustworthiness and limitations of findings | 3 |
| Conflicts of interest | #20 | Potential sources of influence of perceived influence on study conduct and conclusions; how these were managed | 16 |
| Funding | #21 | Sources of funding and other support; role of funders in data collection, interpretation and reporting | 17 |

The SRQR checklist is distributed with permission of Wolters Kluwer © 2014 by the Association of American Medical Colleges. This checklist was completed on 08. April 2018 using http://www.goodreports.org/, a tool made by the EQUATOR Network in collaboration with Penelope.ai

For peer review only

BMJ Open

# Improving the working environment for the delivery of safe surgical care in the United Kingdom: A qualitative cross-sectional analysis


| | |
|---|---|
| Journal: | *BMJ Open* |
| Manuscript ID | bmjopen-2018-023476.R1 |
| Article Type: | Research |
| Date Submitted by the Author: | 14-Oct-2018 |


| | |
|---|---|
| Complete List of Authors: | Baggaley, Alice; Homerton University Hospital NHS Foundation Trust,<br>Robb, Lydia; Royal Infirmary of Edinburgh<br>Paterson-Brown, Simon; Royal Infirmary of Edinburgh<br>McGregor, Richard ; Royal Infirmary of Edinburgh |


| | |
|---|---|
| <b>Primary Subject Heading</b>: | Surgery |
| Secondary Subject Heading: | Medical education and training, Health policy, Surgery, Qualitative research |
| Keywords: | MEDICAL EDUCATION & TRAINING, Organisation of health services < HEALTH SERVICES ADMINISTRATION & MANAGEMENT, SURGERY, QUALITATIVE RESEARCH |


SCHOLARONE™
Manuscripts

# Improving the working environment for the delivery of safe surgical care in the United Kingdom: A qualitative cross-sectional analysis

A. Baggaley[1], L. Robb[2], S. Paterson-Brown[2], R. J McGregor[2]

1. Homerton University Hospital, Hackney, London, UK, E96SR
2. Clinical Surgery, Edinburgh Royal Infirmary, Edinburgh, UK, EH16 4SA

Author correspondence: Richard.McGregor@ed.ac.uk

Word Count: 4048



For peer review only - http://bmjopen.bmj.com/site/about/guidelines.xhtml

**ABSTRACT**

**Objectives:** The aim of this study was to identify current problems and potential solutions to improve the working environment for the delivery of safe surgical care in the United Kingdom.

**Design:** Prospective, questionnaire-based cross-sectional study.

**Setting/Participants:** Following validation, an electronic questionnaire was distributed to postgraduate local education and training board (LETB) distribution lists, the Royal College of Surgeons of Edinburgh (RCSEd) mailing lists and trainee organisations. This consisted of a single open-ended question inviting 5 open-ended responses. Throughout the 13-week study period the survey was also published on a number of social media platforms.

**Results**: A total of 505 responders completed the survey, of which 35% were consultants, 30% Foundation Doctors, 17% speciality trainees, 11% specialty doctors, 5% core trainees, and <1% surgical nurse practitioners. A total of 2238 free text answers detailed specific actions to improve the working environment. These responses were individually coded and then grouped into 9 categories (Staff Resources, Non-Staff Resources, Support, Working Conditions, Communication and Team Work, Systems Improvement, Patient-Centred, Training and Education, Miscellaneous).

**Conclusions**: The results of this study have identified a number of key areas that if addressed, may improve the environment for the delivery of safer surgical care. Common themes that emerged across all grades included: increased front line staff; a return to a "firm" structure to improve team continuity; greater senior support; and improved hospital facilities to help staff rest and recuperate. While unlimited funding remains unrealistic, many of the suggestions could be

For peer review only

implemented in a cost neutral fashion and include insightful ideas for remodeling or restructuring the workforce to improve the efficiency of the surgical team. The findings of this study formed the basis of a set of recommendations published by the RCSEd as a discussion paper.

**Key Words: Training, questionnaire, patient safety**

For peer review only

**Article Summary**

**Strengths and limitations of this study**

1. This study describes the detailed perspectives and opinions of frontline NHS staff regarding current problems and potential solutions for improving the delivery of safe surgical care in the UK at a time of unprecedented strain.
2. The wide distribution of this survey throughout the United Kingdom and response rates across the training/non-training grades helps to mitigate against sub-group bias and generates a depth and richness to the answers.
3. Whilst some groups had higher a number of responders than others this is possibly explained by varying degrees of penetration and distribution across the electronic mailing lists.
4. Despite the brevity of the survey we found a higher than expected incompletion rate (46%), however the demographics of those who did not fully complete the survey were comparable to those that did complete the survey, eliminating a potential completion bias of the respondents.



For peer review only - http://bmjopen.bmj.com/site/about/guidelines.xhtml

5. The extended surgical team is becoming ever more important when considering the multi-disciplinary approach to the delivery of acute surgical services and therefore the limited responses from this group of staff is a weakness.

## INTRODUCTION

Human Factors and Ergonomics (HF&E) is an independent specialty and profession which lies at the intersection of psychology and engineering, with the goal to design working environments to support human performance and improve safety. One of the key principles of HF&E in healthcare is that healthcare professionals do not make errors in isolation, and the working environment should be adjusted in order to support those working within it in order to mitigate these errors[1]. Increasingly the expertise of HF&E specialists are being utilised across the healthcare spectrum, including the domain of surgery, to help us re-design our 'systems' and approaches to improving patient safety[2,3].

While the health and well-being of the work-force underpins a small piece of the overall specialty of HF&E in health care, which in turn ties into patient safety, it is intrinsically linked to team work and team performance[4]. What's more, there is a vast array of literature out-with healthcare, and increasingly within, which focuses upon the critical relationship between non-technical skills, team performance, and improved clinical outcome[5]. The intimate link between human error and patient safety had been placed firmly under the spotlight by a number of key studies that estimate that approximately 10% of all hospital admissions result in an adverse event[6]. This was directly responsible for the introduction of variously titled 'Patient Safety' organisations around the world and the



For peer review only - http://bmjopen.bmj.com/site/about/guidelines.xhtml

development of 'bundles of care' for standardizing protocols for a number of procedures and the World Health Organisation's 19-item Surgical Safety Checklist[7].

The delivery of safe surgical care within the United Kingdom is a complex and multi-faceted issue. To date, there has been limited direct engagement with the stakeholders on the front line of acute surgical services to ascertain what they feel are the current problems in delivering safe surgical care and the potential solutions. The work of this project was initiated through the creation of a short life working group (SLWG) within the RCSEd. The objective of this group was to identify the fundamental factors that could improve the working environment for all those involved in providing safe surgical care. The SLWG aimed to target surgeons and nurse practitioners who were involved in the day to day running of the service. The qualitative data collected was via a cross-sectional survey, from which a number of themes emerged.

## METHODS

### Questionnaire design and distribution

A prospective, cross-sectional, qualitative, online survey was created using 'Survey Monkey'. The survey content was developed by a Royal College of Surgeons of Edinburgh Short Life Working Group (SLWG) (the members of which are detailed in the acknowledgements section). This was approved and validated by the Patient Safety Board, with the explicit aim to elicit as broad a response as possible, from the breadth of the multidisciplinary team.



For peer review only - http://bmjopen.bmj.com/site/about/guidelines.xhtml

The survey was distributed to members of the surgical teams in hospitals across the UK, using the RSCEd mailing list, postgraduate local education and training board (LETB) distribution lists, trainee organisations (e.g. Association of Surgeons in Training), and deanery mailing lists. The utilisation of mailing lists and social media platforms accessible to representatives for foundation trainees, core and speciality trainees provided UK-wide distribution.

The email or social media post detailed the purpose of the survey and included a hyperlink which connected directly to the survey on the 'Survey Monkey' website. Email distribution began in February 2016, with all groups receiving a reminder at 6 and 10 weeks. The survey was open for responses for 13 weeks between 8th February to 14th May 2016. Social media engagement was utilised throughout the aforementioned period.

The survey was entirely voluntary, and no identifiable data was collected or stored. Participants were asked to provide basic demographics including; gender (male, female); grade (consultant, registrar, speciality doctor, core trainee, foundation year trainee, nurse, other); location (England, Wales, Scotland, Northern Ireland); and hospital setting (large city hospital, district general hospital, remote and rural hospital). Participants could select the most relevant from predetermined drop-down options detailed in brackets above.

The questionnaire consisted of one single open-ended question: "In the delivery of safe surgical care, what are the 5 most important things which would improve the workplace environment for you?". Thereafter, sequential white-space boxes allowed for a maximum of 5 free-text responses (up to 4000 characters



For peer review only

each). Survey design dictated mandatory completion of demographics prior to progression onto the free text responses.

### Data analysis

Free text responses were individually coded by two of the authors to allow categorisation. For example, this free-text response from a Consultant; "Improve functionality of electronic patient information systems, particularly prescribing, with access device at every bed space specific to that patient to return ward round assessment to what it was 5 years ago", was assigned the code 'Better IT systems/computers/software' which was subsequently grouped into the Resources category. A minimum of one free text response was required to meet inclusion criteria. The final 92 codes were discussed by the SLWG who agreed upon grouping of the codes into nine broad themes (Figure. 1). The nine themes were as follows: Staff Resources, Non-Staff Resources, Support, Working Conditions, Communication and Team Work, Systems Improvement, Patient-Centred, Training and Education, Miscellaneous. Microsoft Excel (Microsoft, 2010, Redmond, Washington, USA) was used to store the data and generate descriptive statistics.

### Patient Involvement

The questionnaire used in this study was edited and approved by the RCSEd Patient Safety Board, which has lay person representation in addition to medical professionals. The Patient Safety Board also helped devise the key


For peer review only - http://bmjopen.bmj.com/site/about/guidelines.xhtml

recommendations[8] published by RCSEd following the analysis of the survey results (see figure 2).

## RESULTS

A total of 932 people started the survey, however only 505 responders proceeded to complete at least one free text response, resulting in a 54% completion rate. Those 427 remaining responders who did not meet the inclusion criteria of entering at least one free text answer were excluded from further analysis. The 505 participants generated a total of 2238 individual free text responses. The demographics of the respondents are shown in figure 3.

The greatest number of free text responses was generated from consultants and foundation year trainees (801 and 651 respectively). Only 6 nurse practitioners completed any free text responses and their results are not presented here due to insufficient numbers when in comparison to other grades. Figure 4 shows the distribution of responses across each of the nine broad categories, regardless of grade. The vast majority of responses relate to resources, and were further subdivided as either staff or non-staff related resources. The next most frequently recurring responses fell into the communication and teamwork, working conditions, and support categories.

There were a high number of recurring responses throughout the survey, and figure 5 lists the top five from each grade. The need for more staff was an area of concern identified across all grades. This included not only more doctors, but also increased numbers of all members of the surgical team. Interestingly the need


For peer review only - http://bmjopen.bmj.com/site/about/guidelines.xhtml

for more administrative and secretarial staff was mentioned regularly, which may represent the high burden of 'paperwork' in the modern working environment.

*Consultants*

Consultants provided 801 individual free-text responses; over half of these related to resources, both staff and non-staff related (figure 6). The comments regarding non-staff resources included suggestions such as "better information technology systems" (n=28), "more beds" (n=28) and "better availability of imaging/diagnostic services" (n=37). The most frequent individual code pertained to increased staff (n=77), which included medical and surgical axillary staff but particularly noted the need for more nursing staff.

Within the working conditions category over half of the responses pertained to "continuity of team members" and "returning to a 'firm' approach" (n=55). For the support category, consultants mostly referred to support from hospital management (n=25), as opposed to that of senior colleagues as suggested by the more junior grades. Within the communication and teamwork category, consultants suggested better communication skills (n=10), improved teamwork (n=19) and improved morale (n=13) to enhance the working environment. For systems improvements, the need for less bureaucracy (n=20), less focus on service provision (n=11) and a reduction in use of protocols was suggested (n=15). For the patient centred category, the popular responses were continuity of patient care (n=11), and the need to operate on ones' own patients (n=11). This links to the frequently suggested need to return to a 'firm' structure in order to improve continuity of team members.



For peer review only - http://bmjopen.bmj.com/site/about/guidelines.xhtml

*Registrars and Core Trainees*

Collectively this group provided 434 free text responses, with the largest category being non-staff resources (22% compared with 37% of consultant responses) (figure 6). Within the working conditions category, continuity of team members (n=25) and improvement to the rota (n=12) were both popular codes. However, the most frequent code was the suggestion of improvement to facilities such as a hospital mess, increased parking, office space, changing rooms, showers and out of hours catering (n=17). These suggestions were common across all grades. Registrars felt improvements to handovers were important (n=13) - proportionally more so than any other grade – and this may reflect the training posts that the registrars and core trainees hold. Another common theme was that of improvement to appraisals and feedback, as well as an increase in both quality and quantity of teaching (n=20).

*Specialty and Associate Specialty Doctors*

Aligned with the response of the consultant body, around one third of specialty doctors' responses related to non-staff resources such as adequate equipment (n=17) and improved computer systems (n=8). Of all the grades, specialty doctors provided the highest number of responses within the training and education category (figure 6), which mostly related to career progression and access to, and recognition of, training (n=13). Specialty doctors also sought greater support from their seniors as well as hospital management (n=9).


For peer review only - http://bmjopen.bmj.com/site/about/guidelines.xhtml

Subjectively, there appeared to be a sense of frustration at being undervalued within the system. This is of particular importance as progression into training posts declines, making recruitment and retention of specialty doctors even more vital.

*Foundation Year Doctors*

Foundation doctors comprised the second largest group after consultants, with 651 free text responses generated (figure 6). Compared with the other grade groups, foundation year doctors provided the lowest proportional number of responses regarding non-staff resources, but the highest number of responses regarding support (n=120), which frequently involved suggesting more support from their senior colleagues. Compared with consultants, responses from foundation year doctors were much more concerned with communication and teamwork (20% vs. 9%), and suggested that improved handovers, better team cohesion, and clearer plans from their consultants regarding patients might improve the working environment for safe surgical care.

Within the category staff resources, foundation year doctors provided the highest total number of responses (n=129) and tended to seek more staff to support their ward roles, such as phlebotomists, healthcare assistants and physician associates, as well as more 'juniors' to facilitate the staffing of a full rota. Certainly, the recognition of the increasing load of non-clinical ward tasks has led to expansion of the 'wider surgical team', with the recruitment of advance nurse practitioners, prescribing pharmacists and physician associates.



For peer review only - http://bmjopen.bmj.com/site/about/guidelines.xhtml

## DISCUSSION

Many of the themes highlighted by the responses were interconnected and shine a spotlight on the unprecedented strain the National Health Service (NHS) is currently experiencing. For example, it is easy to see how 'rota gaps' left by too few staff can lead to low morale, poor communication, and a lack of continuity of care and teamwork. While unlimited funding remains unrealistic, many of the suggestions from front line staff could be implemented in a relatively cost-neutral fashion. For instance, the use of smart rotas that aim to increase daylight training time and return to a firm structure[9] could improve patient safety, morale and training, and in turn improve recruitment and retention within surgery. Examples of responders' comments can be seen in figure 7. The main core themes highlighted by the survey are discussed below.

### *The Need for Structured Senior Support*

There was a very strong message from the free text answers that better supervision and support for both foundation doctors and speciality trainees would improve overall safety. It is postulated, quite reasonably, that better supervision and support would lead to less stressed juniors who will not only make fewer mistakes, but will have more resilience, higher productivity and less absenteeism[10,11] This in turn will permit better patient care and more meaningful learning to take place in line with the new GMC Standards for Excellence[12]. Communication and a sense of belonging to 'the team' would alleviate such perceptions of lack of support. Both consultants and specialty trainees might



For peer review only - http://bmjopen.bmj.com/site/about/guidelines.xhtml

consider a ‘mid-day catch-up’ with their juniors to provide advice and moral support, phoning the junior doctor in between cases could provide an alternative if seniors were unable to leave theatre to meet at mid-day.

Improving overall support for all trainees is clearly a major factor underpinning both a better working environment but also improved patient safety. This requires a significant change in service delivery so that consultants and specialty trainees are free from all elective activity when ‘on-call’ and can therefore become more involved during the day in the running of the surgical wards and supporting the more junior doctors and other members of the surgical team. Many units now do offer this service and have found that this has allowed a partial return to the old ‘firm’ structure where the same team looks after a group of patients for several days in a row. This requires a significant change in how units and hospitals function, re-organisation of clinics, operating sessions and ward rounds, as well as identification of those surgeons who are not in theatre and might therefore be available to help out with problems on the wards when they arise during the day. The introduction of experienced ‘surgical nurse practitioners’ has greatly facilitated such activities, providing someone who knows the unit system, where senior staff can be found on a daily basis, as well as the unit protocols for management of specific conditions. While such persons require additional funding, those units who have invested in them have reaped rewards in improved efficiency, patient care and trainee support. In some specialties such as Urology, experienced nurses can provide front line urgent clinics, thereby reducing admissions and workload for the trainee doctors[13]. Similarly, consultant led emergency ‘hot clinics’ have been shown to result in a


For peer review only - http://bmjopen.bmj.com/site/about/guidelines.xhtml

significant reduction in emergency admissions and hospital stay, which in turn has an effect on reducing overall workload perception[14].

***The Challenge of Working hours: safety, service, and time for training***

Interestingly, there were very few comments on the number of hours worked. However, linked to the numerous responses surrounding the need for more staff lies the challenge of creating rotas that comply with the European Working Time Regulations while at the same time facilitate excellence in training and safe surgical care. A key document published in March of 2014 highlighted the Impact of the EWTR on the NHS and Health Professionals[15]. As a piece of health and safety legislation, the European Working Time Regulations (EWTR) is intended to reduce fatigue in doctors and improve both their own safety and that of their patients[16]. However, some professional organisations, recognise that it has had consequences, the most important of which is the reduction in the available hours for training within "craft" specialities such as surgery[17]. It is also possible that as a measure to help combat fatigue, the necessary introduction of shift patterns may also have had an unintended consequence on patient care related to a reduction in continuity of care. Training programs find it hard to provide the continuous rest required for the EWTR without utilising full shift rotas. This means that fewer trainees are available but are much busier and have to look after larger numbers of patients. The latter is particularly true in not only acute surgical care environments, but also acute medicine, where the on-call registrar is under constant pressure, another contribution to recruitment difficulties.

Finding the balance between service provision and training has been an interminable challenge for the NHS. Innovative solutions have been suggested and



For peer review only - http://bmjopen.bmj.com/site/about/guidelines.xhtml

the authors point to the highly successful report commissioned by HEE – *Better Training, Better Care*[18]. This programme aimed to improve the quality of training by enabling the key recommendations from Sir John Temple's *Time for Training*[19] and Professor John Collins' *Foundation for Excellence* reports[20]. A pilot project[21] was run in Leeds and York that modified the rotas to maximise the potential time for training, and *"100% of the trainees at Leeds and York have reported more confidence in their work since the pilot and 83% agree, or strongly agree, that they have benefitted from the change in rota. The pilot saw an increase in productivity with weekday activity increasing by 37.7%, weekend activity rising by 29.1% and night shift activity by 22.1%."*

Overall, while it appears clear that patient safety is improved by ensuring that doctors do not work excessive hours over the working week,[22] the current issues relate to arduous night shifts, often with inadequate senior support, that bring their own health-related problems. Studies have shown that staff working night shifts have an increased risk of a road traffic incident whilst driving home[23], as well as less immediate increased risk of type 2 diabetes, cardiovascular disease and some cancers[24]. Other studies have looked into decision making in the context of fatigue, with health workers more likely to make mistakes when sleep deprived[25]; therefore, having a direct impact on patient safety [26].

### *Building a Robust Surgical Workforce for the future*

Following the controversial introduction of the new Junior Doctors contract in 2016, progression into higher training is at an all-time low[27]. A survey in 2017 by the UK Foundation Programme Office found only 42.6% of Foundation Year 2 doctors planned to go directly into training[28]. In the light of the result of


For peer review only - http://bmjopen.bmj.com/site/about/guidelines.xhtml

our survey, building a resilient workforce must be an absolute priority for the future well-being of the NHS. This may be combatted by diversifying our care-provider model to involve the Extended Surgical Team[29], with appropriate skill mix to supplement junior doctors, particularly during peaks of service demands and in areas with chronic shortages in junior medical staffing, allowing junior doctors the time to have more meaningful patient encounters and reduce unnecessary and stressful interruptions in tasks.

***Hospital Facilities***

This study has shown that there remains inadequate access to hot food and appropriate facilities where staff can relax during their breaks (if they are fortunate enough to get them) without meeting patients or their relatives. Lack of a common area, such as a hospital mess, makes it more difficult for consultants and trainees to have "catch up meetings", where advice and moral support can be provided. Improvement in the hospital working environment has been shown to improve safety and quality in hospital care, whilst simultaneous increasing patient satisfaction[30].

**Study Strength and Limitations**

This study focuses on the opinions of NHS staff at the frontline of delivering safe surgical care, who detail the primary problems as well as potential solutions to improve care. The survey was widely distributed throughout the United Kingdom through utilisation of many sources. Response rates across grades alleviates against sub-group bias and provides a broad spectrum of opinions. Whilst some groups had higher response rates than others



For peer review only - http://bmjopen.bmj.com/site/about/guidelines.xhtml

this may be explained by varying degrees of penetration and distribution across the electronic mailing lists. Despite wide distribution of the survey, the incompletion rate was higher than expected (46%). Incompletion of the survey was evenly distributed throughout the demographics therefore there was no indication of potential completion bias of the respondents. Survey design may have been a contributing factor to a low completion rate. The survey may have appeared as a simple tick-box exercise initially, and responders may have felt five free text answers required more time and consideration than they were expecting (especially if completing the survey at work where there are a multitude of distractions). In addition, it may have appeared that it was compulsory to complete all five answers, which it was not, and therefore some responders may have struggled to think of five appropriate answers and terminated the survey for that reason.

Response rates were very limited from nurse practitioners, which leads to the suggestion that distribution to this group was less successful than other grades. This may have been improved by having a nurse practitioner involved in the project - as part of the SLWG - who could then have acted as a representative for nurse practitioners. The concept of the Extended Surgical Team – which includes advanced nurse practitioners, speciality doctors, surgical first assistants, alongside surgeons and surgeons-in-training – is thankfully gaining traction within the UK, and greater efforts in the future need to be devoted to ascertaining what the many not the few think. Historically, doctors and nurses have operated in silos (due to dogma, governance and credentialing) but initiatives such as RCSEd's Faculty of Perioperative Care (FPR) should allow the creation of a true multi-disciplinary team.



For peer review only - http://bmjopen.bmj.com/site/about/guidelines.xhtml

Future work could also involve creating focus groups that connect with specific arms of Government to help inform and influence policymakers working in areas such as workforce wellbeing and recruitment and retention.

**CONCLUSIONS**

The results of the survey were used as a springboard for the development of a RCSEd discussion paper[8] which lists 7 key recommendations (Figure 2). Our findings, alongside these recommendations, form a clear blueprint for Government, policy makers and NHS that could help transform the working environment to improve patient safety, staff morale, and recruitment and retention. To those working in a significant proportion of surgical units, many of these recommendations will not seem surprising. Importantly, this survey provides qualitative evidence about what front line staff feel are the most pressing issues that need to be addressed in order to improve the working environment and create a healthy surgical workforce for the future.

**Figure legend**

**Figure 1: Nine Categories**

Schematic breakdown of the nine broad categories the results were grouped into, from a total of 92 codes generated during analysis.

**Figure 2: Summary of RCSEd Key Recommendations**

These seven recommendations were published in a RCSEd (Royal College of Surgeons of Edinburgh) Discussion Paper titled: *'Improving the Working*



For peer review only - http://bmjopen.bmj.com/site/about/guidelines.xhtml

*Environment for Safe Surgical Care'*, and were proposed in part using the data from this survey.

**Figure 3: Demographics of Responders**

This figure summarises the demographics of those who responded to the survey and met the inclusion criteria (n=505).

**Figure 4: Responses Across Main Categories**

This pie chart demonstrates the total number of responses within each category regardless of grade. With a total of 505 responders, 2238 individual free text answers were generated.

**Figure 5: Top Five Responses According to Grade**

This table demonstrates the top five coded responses per grade. This figure also details the total number of responders within each grade as n=x, from the total of 505 responders who met the inclusion criteria.

**Figure 6: Percentage of category response according to grade**

This figure details the breakdown of response across the categories within each grade. The grades include Consultants, Speciality Doctor, Registrar and Core Trainees together, as well as Foundation Doctors. The numbers detailed within this figure are a percentage of the total number of responses within that specific grade.

**Figure 7: Representative qualitative comments from respondents**



For peer review only - http://bmjopen.bmj.com/site/about/guidelines.xhtml

This figure provides an example of a qualitative comment from each of the nine categories. Each quote is a real example of a response to the survey question.

**Acknowledgements**

The authors are grateful to the members of the SLWG and PSB for their support and involvement in this project.

Short Life Working Group members:

Dr R Aloumi
Miss A Baggaley
Professor S M Griffin
Mr C McIlhenny
Mr S Paterson-Brown
Ms V Dobie
Ms A Hartley
Professor J Hill
Mr R McGregor
Professor R W Parks
Dr L Robb

Patient Safety Board members:

Mr S Paterson-Brown
Ms L Ferguson
Mr T O'Brien
Mr D McArthur
Mr C McIlhenny
Mr A Geraghty
Dr N Maran
Mr G Sunderland
Professor G G Youngson
Professor K Walker
Professor P Peattie
Dr S Russ

**Author Statement:** Survey distributed by AB, LR, SPB and RJM. AB and LR collected the data. AB and LR analysed the data, allocated prospective coding to

For peer review only

each response. AB, LR, SPB and RJM were responsible for compiling the manuscript and approving the final article. AB and LR contributed equally and therefore are joint first authors.

**Funding:** Wellcome Trust

**Conflicts of Interests**: All authors have completed the ICMJE uniform disclosure form at www.icmje.org/coi_disclosure.pdf and declare: this study was commissioned by the Office Bearers of RCSEd and conducted on behalf of the RCSEd; no financial relationships with any organisations that might have an interest in the submitted work in the previous three years. SPB and RJM were RCSEd Council Members at the time of the study. SPB is chair of RCSEd Patient Safety Board at the time of writing.

**Data Sharing Statement:** Respondent-level data are available from the corresponding author at richard.mcgregor@ed.ac.uk. The presented data are anonymised with low risk of identification.

## References

1. Catchpole K. Spreading human factors expertise in healthcare: untangling the knots in people and systems. BMJ Qual Saf. 2013 Oct;22(10):793-7
2. McCulloch P, Morgan L, New S, Catchpole K, Robertson E, Hadi M, Pickering S, Collins G, Griffin D. Combining Systems and Teamwork Approaches to Enhance the Effectiveness of Safety Improvement Interventions in Surgery: The Safer Delivery of Surgical Services (S3) Program. Ann Surg. 2017 Jan;265(1):90-96
3. Yule S, Gupta A, Gazarian D, Geraghty A, Smink DS, Beard J, Sundt T, Youngson G, Mcllhenny C, Paterson-Brown S. Construct and criterion validity testing of the Non-Technical Skills for Surgeons (NOTSS) behaviour assessment tool using videos of simulated operations. Br J Surg. 2018 May;105(6):719-727
4. Dewa CS, Loong D, Bonato S, Trojanowski L. The relationship between physician burnout and quality of healthcare in terms of safety and acceptability: a systematic review. BMJ Open. 2017 Jun21;7(6)



For peer review only - http://bmjopen.bmj.com/site/about/guidelines.xhtml

5. Yule S and Paterson-Brown S. Surgeons' non-technical skills. In: ed Sanchez JA. Patient Safety. Surgical Clinics of North America. Elsevier Inc. 2012 pp37-50
6. Anderson O, Davis R, Hanna GB, and Vincent C. Surgical adverse events: a systematic review. AJS. Elsevier Inc; 2013 Aug 1;206(2):253–62
7. Haynes AB, Weiser TG, Berry WR, Lipsitz SR, Breizat AH, Dellinger EP, Herbosa T, Joseph S, Kibatala PL, Lapitan MC, Merry AF, Moorthy K, Reznick RK, Taylor B, Gawande AA. A surgical safety checklist to reduce morbidity and mortality in a global population. NEJM 2009;360(5): 491-499
8. Paterson-Brown S, McGregor R, Robb L, Baggaley A et al. Improving the working environment for safe surgical care. A discussion paper from the Royal College of Surgeons of Edinburgh. 2017 https://www.rcsed.ac.uk/media/415574/rcsed-working-environment-web3.pdf
9. Professionalism Compliance Analysis Tool (PCAT) for Rotas, Qualitative Semi-structured Interviews of 23 Trainees in Scotland were conducted in 2014 to establish recurring themes of "Good Rotas". Scottish Clinical Leadership Fellows. See appendix 4 for more details.
10. A.J. Oswald et al. Happiness and Productivity. Journal of Labour Economics 2014; 33:(4): 789-822
11. Shanafelt T, Boone S, et al. Burnout and satisfaction with work-life balance among U.S. physicians relative to the general U.S. population. *Arch Intern Med.* 2012;172(18):1377-1385.
12. General Medical Council (GMC). Promoting Excellence: Standards for Medical Education and Training. 2016. www.gmc-uk.org
13. Kaur K, Nevin D, Musgrove G. The Urology Rapid Access Unit (URAU): Tackling Winter Pressures - A 12-month Nurse Led Pilot. British Association of Urological Nurses. 2016
14. Th'ng F, Skouras C, Paterson-Brown A, Ravindran R, Lamb P, de Beaux A, Paterson-Brown S, Mole DJ. Emergency general surgery 'Hot Clinics' reduce admission rates and duration of inpatient stay. Frontline Gastroenterology 2015; 0:1-9. Doi: 10.1136/flgastro-2015-100634
15. The Implementation of the Working Time Directive, and its Impact on the NHS and Health Professionals: Report of the Independent Working Time Regulations Taskforce to the Department of Health (March 2014).
16. Rodriguez-Jareno MC, Demou E, Vargas-Prada S, Sanati KA, Skerjanc A, Reis PG, Helimaki-Aro R, Macdonald EB, Serra C. European Working Time Directive and doctors' health: a systematic review of the available epidemiological evidence. BMJ Open. 2104 Jul 7;4(7)
17. Maisonneuve JJ, Lambert TW, Goldacre MJ. UK doctors' views on the implantation of the European Working Time Directive as applied to medical practice: a quantitative analysis. BMJ Open. 2014 Feb 6;4(2)
18. Better Training, Better Care. Health Education England. http://hee.nhs.uk/wp-content/blogs.dir/321/files/2012/09/Matrix-Knowledge-Better-Training-Better-Care.pdf ).
19. Temple J. *Time for Training.* London: MEE; 2010



For peer review only - http://bmjopen.bmj.com/site/about/guidelines.xhtml

**20.** Collins J. Foundation for excellence. An evaluation of the foundation programme. MEE; 2010.
**21.** Royal College of Surgeons of England. Improving Surgical Training. Proposal for a pilot surgical training programme. Oct 2015 www.rcsengl.ac.uk
**22.** Landrigan CP, Rothschild JM, Cronin JW et al. Effect of reducing interns' work hours on serious medical errors among interns in intensive care units. New England Journal of Medicine, 2004; 351(18): 1838-48.
**23.** Barger LK, Cade BE, Ayas NT. Extended work shifts and the risk of motor vehicle crashes among interns. New England Journal of Medicine. 2004, 352(2): 125-34.
**24.** Proper KI, van de Langenberg D, Rodenburg W et al. The relationship between shift work and metabolic risk factors: a systematic review of longitudinal studies. American Journal of Preventive Medicine. 2016, 50(5): e147-57.
**25.** Parry DA, Oeppen RS, Amin MSA, Brennan PA. Sleep: its importance and the effects of deprivation on surgeons and other healthcare professionals. Br J Oral Maxillofac Surg. 2018 Oct;56(8):663-666
**26.** Mansukhani MP, Kolla BP, Surani S, Varon J, Ramar K. Sleep deprivation in resident physicians, work hour limitations, and related outcomes: a systematic review of the literature. Postgrad Med. 2012 Jul; 124(4):214-9
**27.** Eardley I, Scott H, Wilkingson I. Why a career in surgery is no longer a golden ticket. BMJ 2015;351:h3685
**28.** The Foundation Programme Career Destination Report, 2017. http://www.foundationprogramme.nhs.uk/news/story/f2-career-destination-survey-2017
**29.** Royal College of Surgeons of England. A question of balance. The extended surgical team. April 2016
**30.** Aiken H, Sermeus W, Van den Heede K et al. Patient safety, satisfaction, and quality of hospital care: cross sectional surveys of nurses and patients in 12 countries in Europe and the Unite States. BMJ 2012:344:e1717

For peer review only

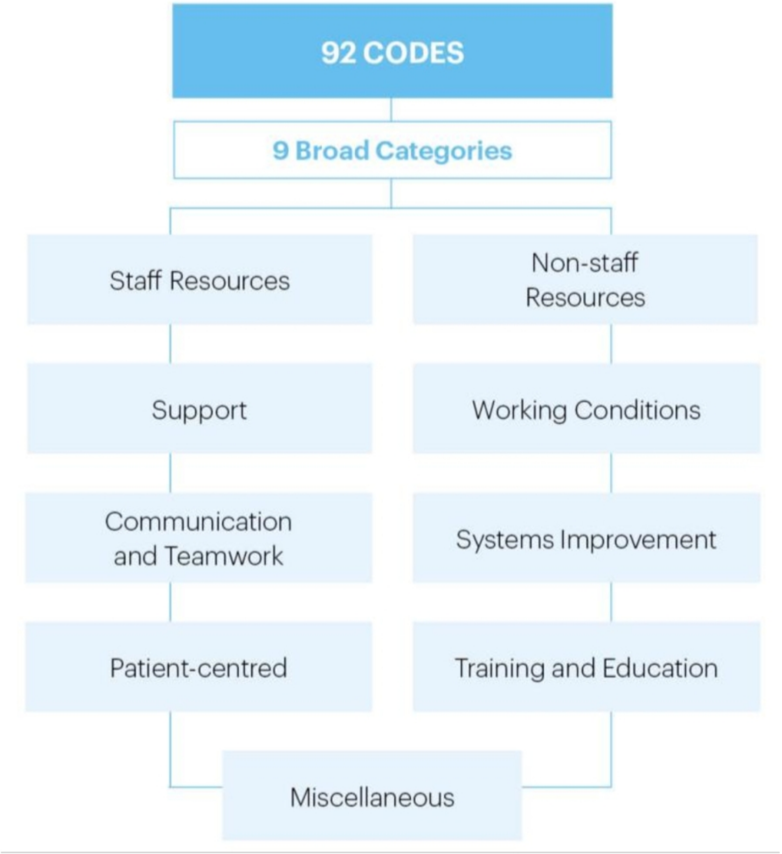


Figure 1: Nine Categories
Schematic breakdown of the nine broad categories the results were grouped into, from a total of 92 codes generated during analysis.

122x126mm (300 x 300 DPI)

| | |
|---|---|
| **Recommendation 1** | Establish structured senior support |
| **Recommendation 2** | Reintroduce the Hospital Mess |
| **Recommendation 3** | Intelligent Design of Rotas |
| **Recommendation 4** | Streamline and reorganize the overall workload to prioritse core clinical duties and creat and integrated multidisciplinary surgical team |
| **Recommendation 5** | Recognise that better training delivers better care |
| **Recommendation 6** | Promote Human Factors training |
| **Recommendation 7** | Support and Training the Trainers |

Figure 2: Summary of RCSEd Key Recommendations
These seven recommendations were published in a RCSEd (Royal College of Surgeons of Edinburgh) Discussion Paper titled: 'Improving the Working Environment for Safe Surgical Care', and were proposed in part using the data from this survey.

106x160mm (300 x 300 DPI)

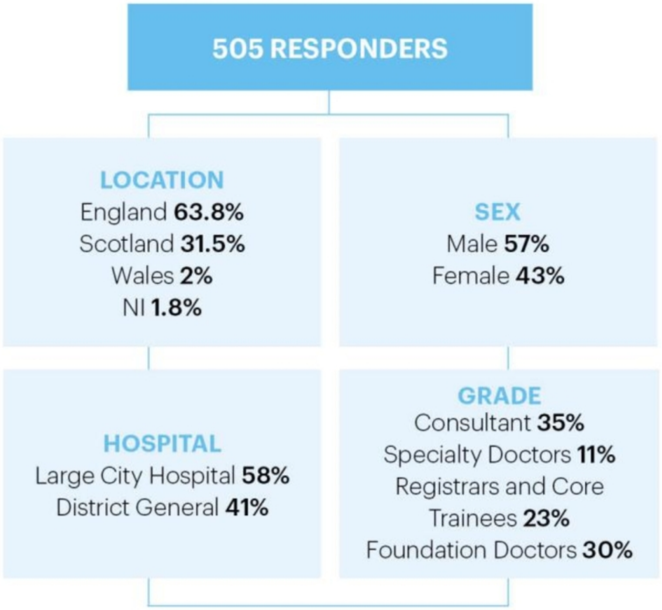


Figure 3: Demographics of Responders

This figure summarises the demographics of those who responded to the survey and met the inclusion criteria (n=505).

133x103mm (300 x 300 DPI)

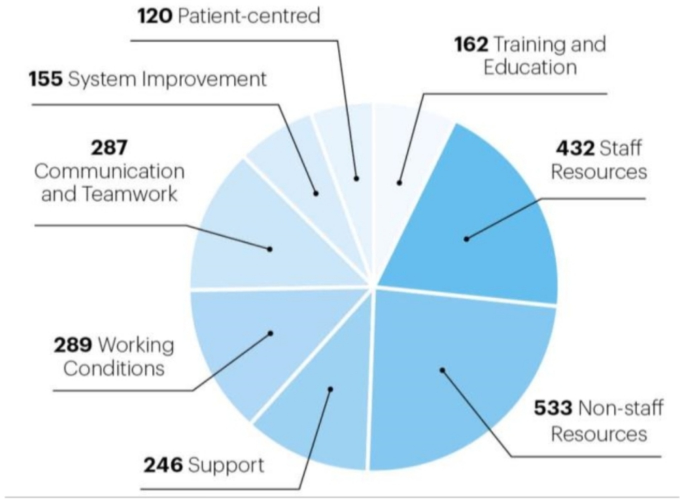


Figure 4: Responses Across Main Categories
This pie chart demonstrates the total number of responses within each category regardless of grade. With a total of 505 responders, 2238 individual free text answers were generated.

136x91mm (300 x 300 DPI)

| Grade | Top 5 Responses |
|---|---|
| **Consultant** (n= 173) | Increased staff ing levels |
| | Return to firm approach |
| | Improved access to imaging/diagnostic services/ interventional radiology |
| | Improved access to emergency theatre |
| | Ring-fenced beds for surgical patients |
| **Specialty Registrar** (n= 83) | **Increased staff ing levels** |
| | Improved facilities |
| | Return to firm approach |
| | Improved IT systems |
| | Improved handover |
| **Core Trainee** (n= 27) | **Increased staff ing levels** |
| | Senior support |
| | Support from other specialities |
| | More time in theatre |
| | Improved facilities |
| **Specialty Doctor** (n= 57) | **Increased staff ing levels** |
| | Senior support |
| | Teaching/training |
| | Adequate equipment |
| | Improved facilities |
| **Foundation Doctor** (n= 151) | **Increased staff ing levels** |
| | Senior support |
| | Teaching/training |
| | Improved IT systems |
| | Better communication |

Figure 5: Top Five Responses According to Grade
This table demonstrates the top five coded responses per grade. This figure also details the total number of responders within each grade as n=x, from the total of 505 responders who met the inclusion criteria.

57x157mm (300 x 300 DPI)

| Categories | Consultant Responses (%) | Speciality Doctor Responses (%) | Registrar and Core Trainee Responses (%) | Foundation Doctor Responses (%) |
|---|---|---|---|---|
| Staff Resources | 15 | 13 | 18 | 20 |
| Non-Staff Resources | 37 | 30 | 22 | 14 |
| Support | 6 | 10 | 9 | 18 |
| Working Conditions | 12 | 10 | 17 | 12 |
| Communication and Teamwork | 9 | 7 | 14 | 20 |
| Systems Improvement | 10 | 8 | 5 | 3 |
| Patient Centred | 7 | 5 | 6 | 3 |
| Training and Education | 3 | 16 | 8 | 10 |
| Miscellaneous | 1 | 1 | 1 | 0 |

Figure 6: Percentage of category response according to grade
This figure details the breakdown of response across the categories within each grade. The grades include Consultants, Speciality Doctor, Registrar and Core Trainees together, as well as Foundation Doctors. The numbers detailed within this figure are a percentage of the total number of responses within that specific grade.

236x156mm (300 x 300 DPI)

| Categories | Example Response |
|---|---|
| Staff Resources | "More value put on support staff such as secretaries and ward clerks to ensure administrative processes work reliably." |
| Non-Staff Resources | "Better out-of-hours access to urgent radiology would improve surgical safety." |
| Support | "We ought to have a senior-led ward round at least once per day." |
| Working Conditions | "There should be on-call facilities so night staff can rest if they are not busy and can sleep in the morning if they are too tired to drive home after night shift." |
| Communication and Teamwork | "Less barriers between senior surgical staff and junior staff : often junior staff can get intimidated and bear the brunt of bad moods which does not allow for safe care of the patient." |
| Systems Improvement | "Consultants to run departments with managers responsible to them - too many clinical decisions are being pushed through by managers who have no knowledge about the consequences if their decisions" |
| Patient Centred | "Junior doctors are always on shifts. They do not provide continuity of care and do not know the patients. the burden is fully on the consultant" |
| Training and Education | "Juniors being given explanations of the surgical procedures which their patients undergo (including having opportunities to go to observe them in theatre where possible), including knowledge of common complications and how to treat them" |

Figure 7: Representative qualitative comments from respondents
This figure provides an example of a qualitative comment from each of the nine categories. Each quote is a real example of a response to the survey question.

57x163mm (300 x 300 DPI)

# Reporting checklist for qualitative study.

Based on the SRQR guidelines.

## Instructions to authors

Complete this checklist by entering the page numbers from your manuscript where readers will find each of the items listed below.

Your article may not currently address all the items on the checklist. Please modify your text to include the missing information. If you are certain that an item does not apply, please write "n/a" and provide a short explanation.

Upload your completed checklist as an extra file when you submit to a journal.

In your methods section, say that you used the SRQR reporting guidelines, and cite them as:

O'Brien BC, Harris IB, Beckman TJ, Reed DA, Cook DA. Standards for reporting qualitative research: a synthesis of recommendations. Acad Med. 2014;89(9):1245-1251.

| | | Reporting Item | Page Number |
|---|---|---|---|
| | #1 | Concise description of the nature and topic of the study identifying the study as qualitative or indicating the approach (e.g. ethnography, grounded theory) or data collection methods (e.g. interview, focus group) is recommended | 1 |
| | #2 | Summary of the key elements of the study using the abstract format of the intended publication; typically includes background, purpose, methods, results and conclusions | 2 |
| Problem formulation | #3 | Description and signifcance of the problem / phenomenon studied: review of relevant theory and empirical work; problem statement | 4 |
| Purpose or research question | #4 | Purpose of the study and specific objectives or questions | 5 |
| Qualitative approach and research paradigm | #5 | Qualitative approach (e.g. ethnography, grounded theory, case study, phenomenolgy, narrative research) and | 6 |

For peer review only

| | | | |
|---|---|---|---|
| | | guiding theory if appropriate; identifying the research paradigm (e.g. postpositivist, constructivist / interpretivist) is also recommended; rationale. The rationale should briefly discuss the justification for choosing that theory, approach, method or technique rather than other options available; the assumptions and limitations implicit in those choices and how those choices influence study conclusions and transferability. As appropriate the rationale for several items might be discussed together. | |
| Researcher characteristics and reflexivity | #6 | Researchers' characteristics that may influence the research, including personal attributes, qualifications / experience, relationship with participants, assumptions and / or presuppositions; potential or actual interaction between researchers' characteristics and the research questions, approach, methods, results and / or transferability | 21 |
| Context | #7 | Setting / site and salient contextual factors; rationale | 6 |
| Sampling strategy | #8 | How and why research participants, documents, or events were selected; criteria for deciding when no further sampling was necessary (e.g. sampling saturation); rationale | 6 |
| Ethical issues pertaining to human subjects | #9 | Documentation of approval by an appropriate ethics review board and participant consent, or explanation for lack thereof; other confidentiality and data security issues | 5 |
| Data collection methods | #10 | Types of data collected; details of data collection procedures including (as appropriate) start and stop dates of data collection and analysis, iterative process, triangulation of sources / methods, and modification of procedures in response to evolving study findings; rationale | 6 |
| Data collection instruments and technologies | #11 | Description of instruments (e.g. interview guides, questionnaires) and devices (e.g. audio recorders) used for data collection; if / how the instruments(s) changed over the course of the study | 6 |
| Units of study | #12 | Number and relevant characteristics of participants, documents, or events included in the study; level of | 8 |

For peer review only

| | | | |
|---|---|---|---|
| | | participation (could be reported in results) | |
| Data processing | #13 | Methods for processing data prior to and during analysis, including transcription, data entry, data management and security, verification of data integrity, data coding, and anonymisation / deidentification of excerpts | 7 |
| Data analysis | #14 | Process by which inferences, themes, etc. were identified and developed, including the researchers involved in data analysis; usually references a specific paradigm or approach; rationale | 7 |
| Techniques to enhance trustworthiness | #15 | Techniques to enhance trustworthiness and credibility of data analysis (e.g. member checking, audit trail, triangulation); rationale | 7 |
| Syntheses and interpretation | #16 | Main findings (e.g. interpretations, inferences, and themes); might include development of a theory or model, or integration with prior research or theory | 8 |
| Links to empirical data | #17 | Evidence (e.g. quotes, field notes, text excerpts, photographs) to substantiate analytic findings | 10 |
| Intergration with prior work, implications, transferability and contribution(s) to the field | #18 | Short summary of main findings; explanation of how findings and conclusions connect to, support, elaborate on, or challenge conclusions of earlier scholarship; discussion of scope of application / generalizability; identification of unique contributions(s) to scholarship in a discipline or field | 12 |
| Limitations | #19 | Trustworthiness and limitations of findings | 17 |
| Conflicts of interest | #20 | Potential sources of influence of perceived influence on study conduct and conclusions; how these were managed | 21 |
| Funding | #21 | Sources of funding and other support; role of funders in data collection, interpretation and reporting | 21 |

The SRQR checklist is distributed with permission of Wolters Kluwer © 2014 by the Association of American Medical Colleges. This checklist was completed on 08. April 2018 using http://www.goodreports.org/, a tool made by the EQUATOR Network in collaboration with Penelope.ai

For peer review only
